# Annotations

**Published:** 1894-01-13

**Authors:** 


### ANNOTATIONS.

### Mince-pie Memoranda.

"THAT was the worst mince-pie I ever tasted in my life. There was no mince-meat in it; what there was was not nice; and the pastry was detestable." Such was the comment of a husband to a wife not many days ago. We will follow some of the physiological consequences of the eating of such a mince-pie. First, the taste was most disagreeable, and the temptation, therefore, was to swallow the mouthful at once. But in that case the food would be neither ground by the teeth, nor mixed, nor charged with saliva and its digestive ferment. The unground, unmixed, and unsalivated morsel is, let us say, passed into the stomach. Now, the fluids of the stomach are useful for chymification, but do not act energetially as substitutes for the grinding teeth. It is not unlikely, therefore, that the unmasticated mince-pie will pass out of the stomach into the bowel unchymified, that is, with the pastry still in lumps and the currants and fruit entire. Arrived in the bowel, there is a decreasing likelihood of grinding operations. The whole fruit remains whole, the lumpy pastry remains lumpy, and there is then commenced an active irritation of the bowel which results in sharp colicky pains, and probably an attack of acute diarrhœa. There are two morals here. The first is: Never eat mince-pies unless they are very nice indeed; the second is: Insist that all food intended to be eaten shall be made palatable and tasty. It is then assured of passing successfully through the first or mouth stage of digestion; and when that stage is very successfully accomplished the battle of complete digestion is almost certainly won.

### Spoilt Patients.

THE Secretary for War announced last week that the principle of the eight hours' day has been adopted in the Ordnance Factories, and that with advantage both to the public service and to the men employed. Let general practitioners of medicine consider this fact a little! Working men are supposed to be the weakest class of all the various classes into which the community is divided; the weakest, that is, in the recognised advantages of birth, brains, education, and money. On the other hand, Governments are, or ought to be, the strongest organisations in the community. Yet it is to its mechanic and operative classes that the strongest Government in the world has yielded an eight hours' working day without any diminution of wages. Now the point we desire to fix the attention of general practitioners upon is the following question and answer. The question is—How has this been done? The answer is—By combination. It is within our knowledge that the health and happiness of a very large proportion of general practitioners are ruined—really and permanently destroyed—by what we venture to call "spoilt patients." There is an enormous class of patients who are "spoilt" by the weakness of general practitioners, just as exacting wives are spoilt by too indulgent husbands, or "petted" children by weak parents. Spoilt patients display the results of their training in going to consult the doctor at unreasonable hours, in calling him out at nights without sufficient justification, in always insisting upon having "the doctor himself" instead of sometimes being satisfied with his partner or qualified assistant, and in being miserably mean about fees. We appeal to the experience of thousands of doctors who have felt the terrible pressure of all these things and are now broken down, or breaking down, in health and spirits in consequence. Is there any cure? Undoubtedly there is; and the cure is in combination—legitimate, loyal, brotherly combination. By combination the half-instructed, and by no means very brotherly, workman has got or is getting all he wants, and much more than many people think he ought to have. "Doctors cannot combine," it is said. Can they not? Then there is no future for the majority of them but increasing competition, increasing drudgery, and increasing poverty. They had better bethink themselves a little more wisely, and see if they cannot find some practicable basis of combination before they succeed in making the lower ranks of general practice altogether intolerable to men of education and reasonable self-respect.

### Medical Workers and Medical Thinkers.

IN all ordinary professions and occupations the thinker takes precedence of the worker, the man of philosophy of the man of practice. To this rule medicine is an exception, and, so far as we can see, the only exception. With us the worker, notwithstanding that his work is generally reported upon by himself, and may in the long run prove to be of no value at all, is nevertheless raised far above the man who may be ten times more competent in general medical knowledge and judgment than himself. It is forgotten that the reporter of cases is in the way of having cases to report, and that he has a money interest in reporting them: the thinker, on the other hand, may not have the cases; and even if he has, he will rarely condescend to waste time and space in the mere piling up of hundreds of separate and similar instances at the cost of large generalisation and philosophical exposition. From this inversion of what is undoubtedly the natural and rational order, medicine has suffered incalculably, and suffered, too, in its very highest interests. For example, although medicine abounds in keen and strong intellects, it is almost impossible, since the death of Sir Andrew Clark, to find a single individual who is capable, by all-round intellectual competency, of holding his own in a public position with other men of similar rank, such as the leaders of the Church and the Bar. In like manner medical literature, although excessive beyond all precedent or necessity in point of quantity, is in most cases of such poor quality that it cannot, except by the exercise of the widest charity, be denominated "literature" at all. Now here is indicated a task which the younger and more philosophically educated medical men of the present day must deal with. The task is to raise medicine to its proper level among the intellectual forces and movements of the time, and also to produce leaders of such philosophic breadth and culture that they shall be able to bear comparison with the greatest leaders of the other cultured professions. Then, and not till then, will the whole body of medical men receive that imperial and general recognition to which by culture and character they are properly entitled. Any profession or nation which holds lightly by its thinkers does so at the cost of its own inevitable degradation.

